# Sirtuin 3 Inhibits Airway Epithelial Mitochondrial Oxidative Stress in Cigarette Smoke-Induced COPD

**DOI:** 10.1155/2020/7582980

**Published:** 2020-09-11

**Authors:** Ming Zhang, Yeli Zhang, Michael Roth, Li Zhang, Rong Shi, Xia Yang, Yali Li, Jie Zhang

**Affiliations:** ^1^Department of Respiratory and Critical Care Medicine, The Second Affiliated Hospital of Xi'an Jiaotong University, Xi'an, Shaanxi, China; ^2^Pulmonary Cell Research & Clinic of Respiratory Medicine, Dept. Biomedicine University of Basel & University Hospital of Basel, CH-4031 Basel, Switzerland; ^3^Department of Urology, The Ninth Hospital of Xi'an, Xi'an, Shaanxi, China

## Abstract

Mitochondrial damage in airway epithelial cells plays an important role in the pathogenesis of chronic obstructive pulmonary disease (COPD). Sirtuin 3 (Sirt3) is a mitochondrial deacetylase regulating mitochondrial function, but its role in the pathogenesis of COPD is still unknown. The aim of the present study was to investigate the effect of Sirt3 on airway epithelial mitochondria in cigarette smoke-induced COPD. Our present study has shown serious airway inflammation, alveolar space enlargement, and mitochondrial damage of the airway epithelium in COPD rats. Compared to the control rats, Sirt3 protein expression was significantly decreased in the airway epithelium and lung tissue homogenate from COPD rats. In airway epithelial cells (BEAS-2B), cigarette smoke extract (CSE) treatment significantly decreased mRNA and protein expression of Sirt3 and manganese superoxide dismutase (MnSOD), as well as MnSOD activity in a concentration and time-dependent manner. Sirt3 siRNA further significantly intensified the decreases in MnSOD expression and activity and aggravated mitochondrial oxidative stress and cell injury when airway epithelial cells were treated with 7.5% CSE. In contrast, Sirt3 overexpression significantly prevented the decrease of MnSOD expression and activity and improved mitochondrial oxidative stress and cell injury in CSE-treated airway epithelial cells. These data suggest that Sirt3 inhibits airway epithelial mitochondrial oxidative stress possibly through the regulation of MnSOD, thereby contributing to the pathogenesis of COPD.

## 1. Introduction

Chronic obstructive pulmonary disease (COPD) is characterized by poorly reversible and progressive airflow limitation. Currently, about 250 million patients suffer from COPD worldwide [1, 2]. The overall prevalence of spirometry-defined COPD in China was 8.6%, accounting for 99.9 million people with COPD [3]. Cigarette smoking is assumed to be the main cause of COPD, but the etiology and pathogenesis of this disease are still unclear [4].

The airway epithelium functions as a barrier to defend the lung against inhaled antigens and pathogens. Alterations of the airway epithelium function have been proposed to play an important role in the pathogenesis of chronic airway inflammatory disease, including COPD [5]. In this regard, cigarette smoke-induced oxidative stress of the airway epithelium is implicated to initiate the pathogenesis in COPD [6]. Mitochondria are a major source of oxygen radicals and a target for their damaging effects. Therefore, mitochondrial oxidative stress of the airway epithelium appears to be a cause rather than a consequence of COPD [7].

Sirtuins were first identified as nicotinamide adenine dinucleotide- (NAD^+^-) dependent type III histone deacetylases [8]. Mammalian sirtuins target not only histones in the nucleus but also other proteins in the cytoplasm and mitochondria [9]. Seven members of sirtuins have been reported in mammals, and they are widely expressed in normal tissues [9]. Sirtuin (Sirt) 1, 6, and 7 are primarily localized within the nucleus; Sirt2 is present in the cytoplasm, while Sirt3, 4, and 5 are mainly localized in the mitochondria. Sirt3 is a primary mitochondrial deacetylase and involved in fatty acid *β*-oxidation, amino acid metabolism, and electron transport chain [10]. It has been reported that caloric restriction as well as exercise promotes neuroprotection and extends lifespan in mammals, through the protective effect of Sirt3 on mitochondria [10, 11].

Furthermore, Sirt3 is involved in mitochondrial reactive oxygen species (ROS) scavenging and antioxidant defense [12]. Sirt3 knockout mice are prone to metabolic syndrome [13], cancer [14], and cardiac failure [15], which are all linked to the high levels of cellular ROS. Sirt3 overexpression significantly decreased the levels of ROS and reduced mitochondrial oxidative stress in a feedback mechanism [16]. This antioxidant effect of Sirt3 was mediated through the activation of manganese superoxide dismutase (MnSOD) [17], which acts as a mitochondrial detoxification enzyme. Sirt3 deacetylates lysine residues 53, 68, 89, and 122 of MnSOD, and thereby increases its enzymatic activity [18–20]. Moreover, Sirt3 has been shown to interact with FOXO3a in mitochondria [21]. Sirt3 overexpression increased FOXO3a DNA-binding activity and then stimulated the gene expression of MnSOD [21]. Thus, Sirt3 inhibits mitochondrial oxidative stress through upregulating MnSOD expression and deacetylation.

However, the role of Sirt3 in airway epithelial mitochondrial damage in COPD remains unknown. Therefore, this study investigated the protein expression of Sirt3 in airway epithelium of COPD rats and further explored the effects of Sirt3 on airway epithelial mitochondrial oxidative damage induced by cigarette smoke extract (CSE).

## 2. Materials and Methods

### 2.1. Materials

Septwolves cigarettes (tobacco type of tar: 10 mg, nicotine content: 0.8 mg, carbon monoxide fumes: 12 mg) were from the China Tobacco Fujian industry limited liability company (Xiamen, China). The human bronchial epithelial cell line (BEAS-2B) was obtained from the Shanghai Cell Bank of the Chinese Academy of Sciences (Shanghai, China). Sirt3, MnSOD, and *β*-actin antibodies were purchased from Cell Signaling Technology (Beverly, MA, USA). Sirt3 siRNA and Sirt3 plasmid were purchased from GenePharma Co. Ltd. (Shanghai, China).

### 2.2. COPD Rat Model

The animal study protocol was approved by the Institutional Animal Research and Ethics Committee of Xi'an Jiaotong University. Fourteen male Sprague-Dawley rats were purchased from the Animal Center of Xi'an Jiaotong University and divided into the control (*n* = 6) and COPD model (*n* = 8) groups. The methods to generate the COPD model were described previously [22]. In brief, rats in the COPD model group received intratracheal instillation of lipopolysaccharide (1 mg/mL, 0.2 mL) on days 1 and 15 and were exposed to smoke from 6 commercial unfiltered cigarettes for 30 min, twice a day, for a total of 60 days except days 1 and 15. Control rats were exposed to the room air with the same procedure.

### 2.3. Blood Gas Analysis and Cytology of Bronchoalveolar Lavage Fluid (BALF)

At day 61, all rats were weighted and anesthetized by intraperitoneal injection of chloral hydrate (300 mg/kg). Blood samples were collected from the abdominal aorta, and partial pressure of oxygen and carbon dioxide in the arterial blood (PaO_2_ and PaCO_2_) were immediately determined by blood gas analyzer (Radiometer ABL800, Denmark).

BALF cytology was studied by Wright staining, which is widely used in the differential cell counts in BALF [23]. In brief, three mL of sterile phosphate-buffered saline (PBS) was instilled 3 times via the tracheal cannula and recovered by gentle manual aspiration. The 3 lavage fractions were centrifuged at 2000 g for 10 min at 4°C, and the cell pellets were resuspended in 1 mL of PBS. The total number of cells was counted using a hemocytometer, and differential cell counts were calculated by Wright staining.

### 2.4. Histopathology

The right upper lobe tissues were fixed in 4% paraformaldehyde, paraffin embedded, and finally stained with hematoxylin-eosin (HE). Alternatively, fresh tissue from the left upper lobe bronchus was fixed with 2.5% glutaraldehyde, followed by 1% perosmic acid and dehydrated in an ethanol series. Ultrathin tissue sections were placed on 400 mesh grids and double-stained with uranyl acetate lead citrate and analyzed by transmission electron microscope (HITACHI-H7650, Tokyo, Japan).

### 2.5. Immunohistochemistry Analysis

The tissue sections were deparaffinized, and antigen retrieval was performed by citrate buffer incubation. The sections were then incubated in 3% H_2_O_2_ for 5 min, blocked for 20 min with 20% normal goat serum, and incubated with the Sirt3 antibody (1 : 200) overnight at 4°C. The slides were then incubated with diluted biotinylated secondary antibody for 30 min. Antibody binding was visualized by ABC Elite kit (Boster) with DAB and counterstained with hematoxylin. The staining intensity was scored as either absent (0), weak (1), moderate (2), or strong (3), and the percentage of stained cells were quantified as 0 (<5%), 1 (5-25%), 2 (26-50%), 3 (51-75%), or 4 (76-100%). The multiplication of intensity and percentage scores was used as the final protein staining score and represented the protein expression of Sirt3.

### 2.6. CSE Preparation

Septwolves cigarettes were used to prepare CSE as described earlier [24]. Briefly, a total of 300 mL cigarette smoke was collected by a syringe apparatus and bubbled in 10 mL RPMI medium. The crude CSE was adjusted pH to 7.4 and filtered through a 0.22 *μ*m filter. This solution was defined as 100% CSE, and working concentration was prepared by dilution with culture medium.

### 2.7. Cell Culture and Transient Transfection

Human bronchial epithelial cells (BEAS-2B) were cultured in RPMI 1640 medium supplemented with 10% fetal bovine serum (Gibco) in a humidified incubator under 5% CO_2_ at 37°C. The cells were transfected with Sirt3 siRNA or Sirt3 plasmid using Lipofectamine 2000 (Invitrogen) according to the manufacturer's protocol. Negative control siRNA and empty plasmid vector were used in parallel.

### 2.8. Real-Time PCR

Total mRNA was extracted and reverse transcription was carried out using an RT-PCR kit (TaKaRa), and finally, the mRNA expression of Sirt3 and MnSOD was determined by real-time PCR (TaKaRa). The primers were as follows: 5′-ACCCAGTGGCATTCCAGAC-3′ (forward) and 5′-GGCTTGGGGTTGTGAAAGAAG-3′ (reverse) for Sirt3; 5′-AACGTCACCGAGGAGAAGTA-3′ (forward) and 5′-TGATAGCCTCCAGCAACTCT-3′ (reverse) for MnSOD; and 5′-AGCGAGCATCCCCCAAAGTT-3′ (forward) and 5′-GGGCACGAAGGCTCATCATT-3′ (reverse) for *β*-actin. Results were expressed as fold differences relative to the level of *β*-actin using the 2^-*△△*CT^ method.

### 2.9. Western Blot

Lung tissue and BEAS-2B cells were homogenized in RIPA lysis buffer containing protease inhibitors. Protein concentration was determined by a BCA protein assay kit (Beyotime, China). Total protein (25 *μ*g) was subjected to sodium dodecyl sulfate-polyacrylamide gel electrophoresis and transferred onto a polyvinylidenedifluoride membrane. The membranes were then incubated with specific antibodies against Sirt3 (1 : 1000), MnSOD (1 : 1000), or *β*-actin (1 : 1000) at 4°C overnight. Protein bands were visualized by incubation of membranes with species-specific secondary HRP-conjugated antibody by chemiluminescence substrate (Thermo Scientific, USA). Results were expressed as fold increase over control.

### 2.10. MnSOD Activity Measurement

MnSOD activity was measured by spectrophotometry following the commercial kit manual (Nanjing Jiancheng Bioengineering Institute, Nanjing, China), and the results were expressed as units per milligram of protein.

### 2.11. Mitochondrial Membrane Potential (MMP) Determination

The level of MMP was assessed by staining the cells with JC-1 fluorescence dye (Beyotime, China). JC-1 forms J-aggregates emitting red fluorescence at 590 nm in healthy mitochondria and J-monomers emitting green fluorescence at 490 nm in depolarized mitochondria. The emission intensity at both wavelengths was observed and evaluated by a fluorescent microscope (Olympus, Tokyo, Japan), and the results were expressed as the ratio of red to green fluorescent intensity.

### 2.12. Detection of Mitochondrial ROS

MitoSOX Red (Invitrogen), a live-cell permeant dye that rapidly and selectively targets mitochondria, was used to measure the levels of mitochondrial ROS. Fluorescent intensity was detected by a fluorescent microscope (Olympus, Tokyo, Japan) at 510 nm excitation and 580 nm emission wavelengths and further quantified using the Image-Pro Plus 6.0 software.

### 2.13. Apoptosis Assay

The cell surface exposure of phosphatidylserine and plasma membrane disruption was evaluated by staining with annexin V-FITC and PI according to the manufacturer's protocol (KeyGEN BioTECH, China). The cells were analyzed by FACSCalibur flow cytometer (Becton Dickinson GmbH, Heidelberg, Germany). The apoptosis rate was expressed as the percentage of annexin V-FITC-positive cells to the total cells.

### 2.14. Cell Viability Assay

Cell viability was determined by the conventional 3-(4,5-dimethylthiazol-2-yl)-2,5-diphenyltetrazolium bromide (MTT) assay and expressed as a percentage of the control group.

### 2.15. Statistical Analysis

All data are presented as mean ± standard deviation (SD) and were analyzed using SPSS 16.0 software. The statistical analysis was performed by Student's *t* test for the comparison between control and model groups. The effects of CSE on airway epithelial cells were analyzed by one-way analysis of variance test and LSD test. A *P* value < 0.05 was considered as significant.

## 3. Results

### 3.1. COPD Rat Model

**Figure 1:**
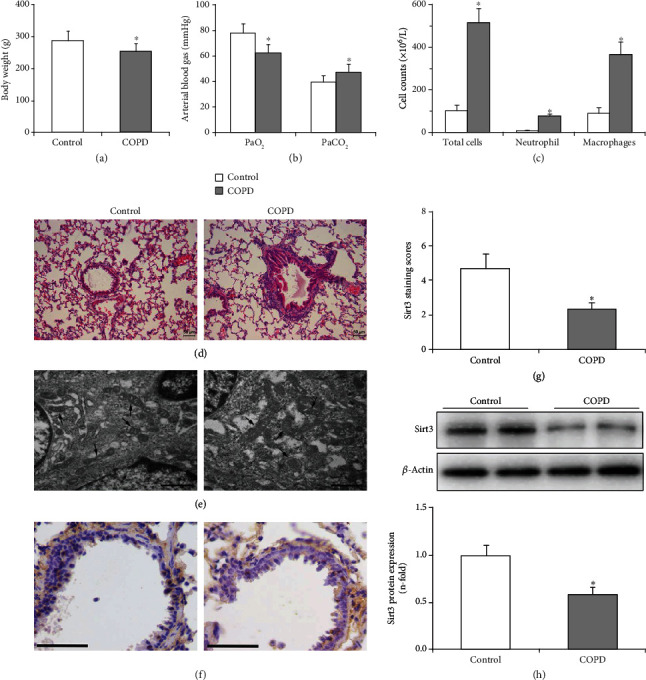
Decreased Sirt3 protein expression in airway epithelium of COPD rats is associated with airway epithelial mitochondrial damage. (a) Body weight of COPD rats was significantly decreased compared with the healthy control rats. (b) Arterial blood gas analysis results showed that PaO_2_ significantly decreased and PaCO_2_ was increased in COPD rats compared to control rats. (c) The numbers of total cells, neutrophils, and macrophages in BALF from the COPD group were significantly higher than those in the control group. (d) Representative HE staining of lung tissue sections (×200). (e) Representative electron microscope images of the ultrastructure alterations of airway epithelial cells in rats (×30000). COPD rats showed swollen mitochondria with fractured or dissolved cristae as indicated by the arrows. The scale bar represents 1 *μ*m. (f) Representative immunohistochemistry image for Sirt3 in rat airway epithelium. The scale bar indicates 50 *μ*m. (g) Quantification analysis of Sirt3 protein expression in lung tissue sections of COPD and control rats. The Sirt3 staining score is defined as the multiplication of staining intensity and percentage of stained cells, and the results showed that Sirt3 protein expression was significantly decreased in COPD rats compared to control rats. (h) The protein expression of Sirt3 in lung tissue homogenate was determined by Western blot. A representative blot is shown in the upper panel, and the quantification analysis shown in the lower panel displayed that Sirt3 expression was significantly decreased in COPD rats. Data are shown as mean ± SD (*n* = 6 and 8, respectively). ^∗^*P* < 0.05 compared to the control group.

Rats in the control group were active and had a good appetite, steady breathing, and few respiratory secretions. In contrast, COPD rats showed inanimate behavior and had an edge off appetite and increased respiratory secretions. Body weight of COPD rats was lower than that in the control group (254 ± 25.3 g versus 288 ± 30.3 g, *P* < 0.05, Figure 1(a)). When compared to control rats, arterial blood analysis showed that COPD rats had lower PaO_2_ (62.6 ± 6.30 mmHg versus 77.7 ± 7.74 mmHg, *P* < 0.05) and higher PaCO_2_ (47.3 ± 6.76 mmHg versus 39.8 ± 5.00 mmHg, *P* < 0.05) (Figure 1(b)). Counting of inflammatory cells in BALF showed that the number of total cells in COPD rats was significantly increased compared to control rats (519 ± 64.5 × 10^6^/L versus 105 ± 24.2 × 10^6^/L, *P* < 0.01, Figure 1(c)). Further differential cell counts indicated that the numbers of neutrophils and macrophages in BALF from COPD rats were both significantly higher than those in the control rats (*P* < 0.01, Figure 1(c)).

### 3.2. Pathological Changes in Lung Tissue

Rat lung tissue sections were observed under the light microscope, and representative images are shown in Figure 1(d). HE staining showed an orderly arranged epithelium with no inflammatory cell infiltration and no gland hyperplasia in control rats. In contrast, shedding of airway epithelial cells, hypertrophy of goblet, and glandular cells, as well as inflammatory cell infiltration were observed in the lungs of COPD rats. Compared to control rats, the enlargement of the alveolar space, alveoli damage, and alveoli fusion into bullae was observed in the lungs of COPD rats.

Analysis by electron microscopy revealed morphological changes of the airway epithelium ultrastructure in rats (Figure 1(e)). The airway epithelial cells of control rats displayed normal cilia in neat rows, preserved mitochondria, maintained endoplasmic reticulum, and integrity nuclei with homogeneous chromatins. While the ultrastructural alterations of airway epithelial cells in the COPD rats were significant, including disarranged cilia, swollen mitochondria with fractured or dissolved cristae, dilated endoplasmic reticulum, and irregular cell nuclei.

### 3.3. Protein Expression of Sirt3 in Lung Tissue

Sirt3 protein expression in the airway epithelium was determined by immunohistochemistry, and the results revealed that its expression (brown staining) in airway epithelial cells was significantly decreased in COPD rats compared to the control group (Figure 1(f)). Furthermore, quantitative image analysis indicated that Sirt3 protein expression in COPD rats was significantly reduced to 0.49-fold compared to the control rats (*P* < 0.01, Figure 1(g)). Sirt3 protein expression in the lung tissue homogenate was determined by Western blot, and the results confirmed that its expression was significantly lower in COPD rats than in control rats (*P* < 0.01, Figure 1(h)).

### 3.4. CSE Decreased Sirt3 and MnSOD Expression in Airway Epithelial Cells

**Figure 2:**
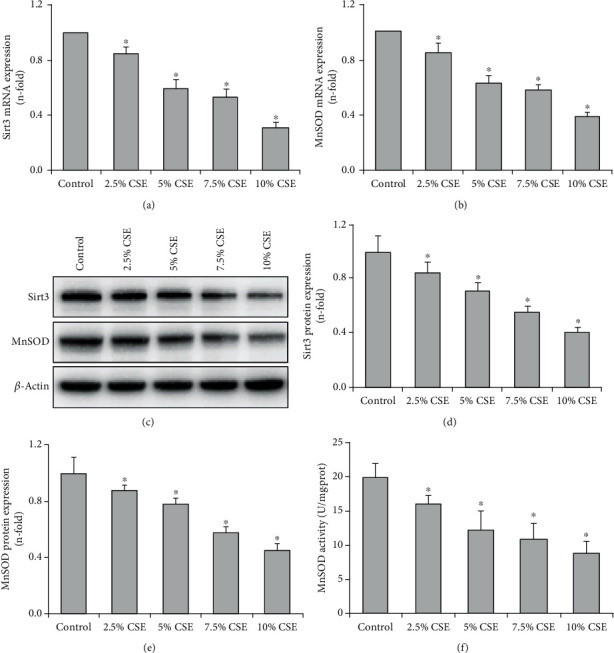
CSE decreased Sirt3 and MnSOD expression and MnSOD activity in a concentration-dependent manner in airway epithelial cells. When airway epithelial cells were stimulated with CSE (2.5-10%) for 24 h, real-time PCR showed that the mRNA expression of Sirt3 (a) and MnSOD (b) was decreased in a concentration-dependent manner. The protein expression of Sirt3 and MnSOD was determined by Western blot, and a representative blot is shown in part (c). Quantification analysis displayed that the expression levels of Sirt3 (d) and MnSOD (e) were significantly decreased in a concentration-dependent manner when airway epithelial cells were stimulated with CSE (2.5-10%) for 24 h. After 24 h stimulation with CSE, MnSOD activities in airway epithelial cells were gradually decreased at the indicated concentration (f). All statistical data were obtained from three independent experiments and presented as mean ± SD. ^∗^*P* < 0.05 versus control group.

**Figure 3:**
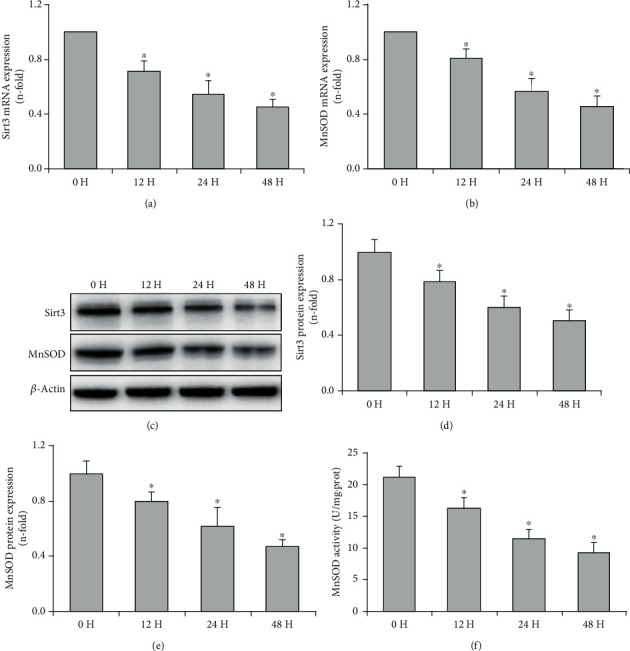
CSE decreased Sirt3 and MnSOD expression and MnSOD activity in a time-dependent manner in airway epithelial cells. After the stimulation with 7.5% CSE, real-time PCR showed that Sirt3 (a) and MnSOD (b) mRNA expressions were decreased in a time-dependent manner. A representative blot of Sirt3 and MnSOD expressions determined by Western blot is shown for each condition (c), and quantification analysis displayed that the expression levels of Sirt3 (d) and MnSOD (e) were decreased in a time-dependent manner when BEAS-2B cells were stimulated with 7.5% CSE at the indicated time points. MnSOD activities were gradually decreased when BEAS-2B cells were incubated with 7.5% CSE for 0-48 h (a). All statistical data were obtained from three independent experiments and presented as mean ± SD. ^∗^*P* < 0.05 compared to the cells treated with CSE for 0 h.

The effect of CSE on Sirt3 and MnSOD expression in airway epithelial cells was determined by real-time PCR and Western blot. The results showed that the mRNA and protein expressions of Sirt3 and MnSOD were significantly decreased in a concentration-dependent manner when airway epithelial cells were stimulated with 2.5-10% CSE for 24 h (*P* < 0.05, Figures 2(a)–2(e)). Then, the airway epithelial cells were exposed to 7.5% CSE at different time points, and the mRNA and protein expression of Sirt3 and MnSOD were also decreased in a time-dependent manner as shown in Figures 3(a)–3(e) (*P* < 0.05). In addition, enzyme activity assay displayed that MnSOD activities were significantly decreased in a concentration and time-dependent manner when airway epithelial cells were stimulated with CSE (*P* < 0.05, Figures 2(f) and 3(f)).

### 3.5. Sirt3 Increased MnSOD Expression and Inhibited Mitochondrial Oxidative Stress in Airway Epithelial Cells Exposed to CSE

**Figure 4:**
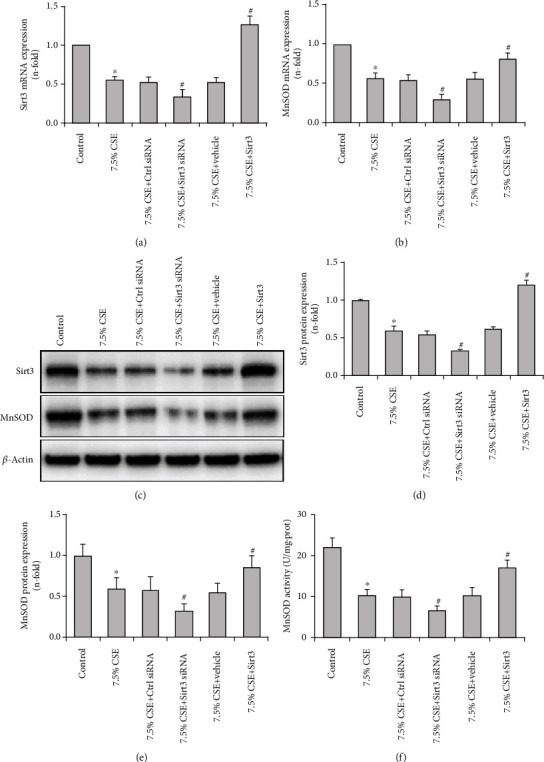
Sirt3 regulated the expression and activity of MnSOD in airway epithelial cells exposed to CSE. Real-time PCR showed that the declines in Sirt3 (a) and MnSOD (b) expressions were significantly intensified by Sirt3 siRNA and improved by Sirt3 overexpression in CSE-treated BEAS-2B cells. A representative blot of Sirt3 and MnSOD expressions determined by Western blot is shown for each condition (c). Further quantification analysis revealed that the deceases in protein expression of Sirt3 (d) and MnSOD (e) were significantly aggravated by Sirt3 siRNA and reversed by Sirt3 overexpression when BEAS-2B cells were stimulated with 7.5% CSE. The decline in MnSOD activity was significantly intensified by Sirt3 siRNA and prevented by Sirt3 overexpression in BEAS-2B cells exposed to 7.5% CSE (f). All values are shown as mean ± SD from three independent experiments. ^∗^*P* < 0.01 versus control group, and ^#^*P* < 0.05 versus 7.5% CSE-treated group.

In order to demonstrate the role of Sirt3 in CSE-induced mitochondrial injury in airway epithelial cells, Sirt3 expression was knocked down by siRNA or upregulated by a Sirt3 expression vector. In BEAS-2B cells stimulated with 7.5% CSE, silencing of Sirt3 further reduced Sirt3 and MnSOD expression (*P* < 0.01, Figures 4(a)–4(e)). In contrast, transfection with the Sirt3 expression vector significantly upregulated Sirt3 expression and prevented the CSE-induced decrease of MnSOD expression in BEAS-2B cells (*P* < 0.05, Figures 4(a)–4(e)). Furthermore, the decrease of MnSOD activity was aggravated by Sirt3 siRNA and improved by Sirt3 overexpression when airway epithelial cells were exposed to 7.5% CSE (*P* < 0.05, Figure 4(f)).

**Figure 5:**
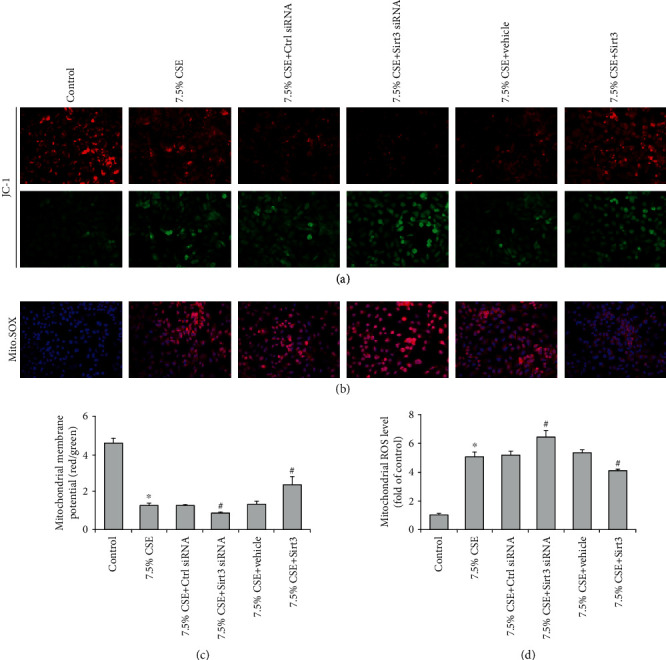
Sirt3 inhibited airway epithelial mitochondrial oxidative stress induced by CSE. Airway epithelial cells were stained with JC-1 or Mito. SOX fluorescence dye and the fluorescence intensities determined by fluorescent microscope represented the levels of mitochondrial membrane potential (a) and mitochondrial ROS (b). Quantitative analysis showed CSE decreased mitochondrial membrane potential level (c) and increased mitochondrial ROS content (d), and the alterations were aggravated by Sirt3 siRNA and attenuated by Sirt3 overexpression in CSE-treated BEAS-2B cells. Results are expressed as mean ± SD from three independent experiments. ^∗^*P* < 0.01 versus control group, and ^#^*P* < 0.05 versus 7.5% CSE-treated group.

**Figure 6:**
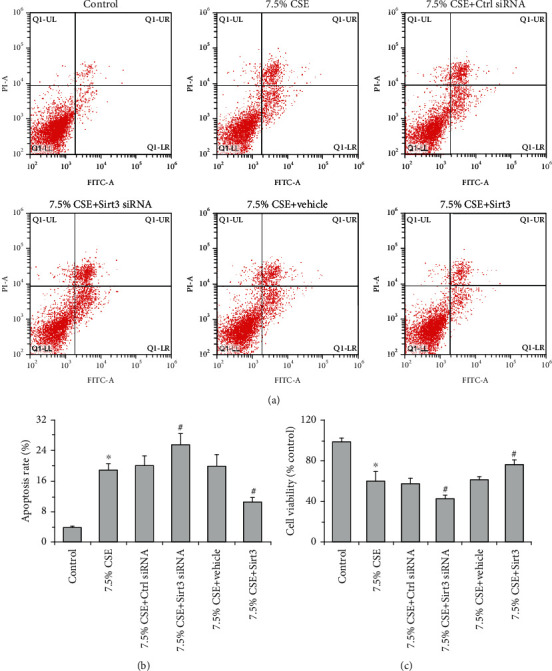
Sirt3 improved airway epithelial cell injury induced by CSE. Representative images of cell apoptosis determined by flow cytometry are shown for each condition (a). The increases in cell apoptosis rate were further elevated by Sirt3 siRNA but inhibited by Sirt3 overexpression when BEAS-2B cells were stimulated with 7.5% CSE (b). MTT assay showed that the decreases in cell viability were further intensified by Sirt3 siRNA and prevented by Sirt3 overexpression in CSE-treated BEAS-2B cells (c). Data are expressed as mean ± SD from three independent experiments. ^∗^*P* < 0.01 versus control group, and ^#^*P* < 0.05 versus 7.5% CSE-treated group.

Mitochondrial oxidative stress of airway epithelial cells was assessed by MMP and mitochondrial ROS levels (Figure 5). The results showed that the treatment with 7.5% CSE significantly decreased the MMP level but increased mitochondrial ROS content. CSE-induced mitochondrial oxidative stress was further aggravated by Sirt3 siRNA transfection, but attenuated by Sirt3 overexpression (*P* < 0.01, Figure 5). Moreover, 7.5% CSE significantly increased cell apoptosis rate and decreased cell viability determined by flow cytometry and MTT, respectively (*P* < 0.01, Figure 6). Compared to the airway epithelial cells treated with 7.5% CSE, Sirt3 siRNA transfection notably increased cell apoptosis and decreased cell viability (*P* < 0.01, Figure 6). In contrast, the two cell injury indexes (cell apoptosis and viability) were significantly improved in airway epithelial cells that overexpressed Sirt3 compared to cells treated with 7.5% CSE (*P* < 0.01, Figure 6).

## 4. Discussion

The presented data demonstrate that the protein expression of Sirt3 in the airway epithelium of COPD rats was significantly decreased and was associated with severe mitochondrial damage. In vitro experiments showed that Sirt3 regulated the expression and enzyme activity of MnSOD, and thereby inhibited CSE-induced mitochondrial oxidative stress in human airway epithelial cells.

COPD is a life-threatening lung disease, which is currently the fourth leading cause of morbidity and mortality worldwide, without curative treatment available. The pathogenesis of COPD has been largely investigated, while the underlying molecular mechanism and pathophysiology remain elusive. Airway epithelial cell functions as the first defense barrier against cigarette smoke or inhaled environment pollutants. Thus, the impairment of airway epithelial cell function may initiate the pathogenesis of COPD [25]. It is well known that mitochondrial dysfunction leads to airway epithelial cell damage [26], thus airway epithelial mitochondrial damage is involved in the pathogenesis of COPD. Structural and functional changes of the mitochondria in the epithelium of COPD lungs are potentially the consequence of long-term exposure to cigarette smoke [27]. In a previous study, we demonstrated that CSE induced mitochondrial damage of airway epithelial cells, including MMP loss, mitochondrial ROS increase, and ATP decline [24]. In addition, mitophagy promotes cigarette smoke-induced mitochondria and airway epithelial cell damage [28, 29]. Therefore, mitochondrial damage in the airway epithelium has been recognized as a contributing factor to the pathogenesis of COPD.

Sirt3 is mainly expressed in mitochondria and closely associated with mitochondrial damage [30]. It has been reported that Sirt3 regulates mitochondrial function through the deacetylation of several metabolic and respiratory enzymes [31]. The role of Sirt3 has been investigated in several diseases, such as Alzheimer's disease [32], cardiovascular diseases [33], and renal diseases [34]. In addition, Sirt3 is involved in the physiological process of aging [31], and its expression in skeletal muscle was reduced by 50% in elderly individuals compared to young subjects [35]. However, the role of Sirt3 in mitochondrial damage of the airway epithelium during the progression of COPD is still unknown.

In COPD rats, Sirt3 expression was significantly decreased in skeletal muscle tissues [22], but it is unknown whether Sirt3 expression is also decreased in the airway epithelium of COPD lungs. Therefore, the protein expression of Sirt3 in the airway epithelium of COPD rats has been further studied in this study. Firstly, a rat model of COPD has been established according to our previous experiments [22, 36]. The result of blood gas analysis showed that COPD rats suffered from hypoxemia and hypercapnia, and histopathological changes were consistent with the characteristics of COPD. BALF cytology of the presented COPD rat model confirmed the alterations of inflammatory cells in BALF from COPD rats [37]. Sirt3 protein expression in the rat lung tissue determined by immunohistochemistry and Western blot demonstrated that Sirt3 expression in the airway epithelium of COPD rats was significantly decreased and was associated with mitochondrial damage in the airway epithelium. These data support the hypothesis that Sirt3 activation might protect against mitochondrial damage of the airway epithelium in COPD.

In order to further explore the effect of Sirt3 on airway epithelial mitochondria in COPD and its underlying mechanism, the role of the Sirt3-MnSOD signaling pathway in CSE-treated airway epithelial cells was studied. Our study has demonstrated that CSE significantly decreased the mRNA and protein expression of Sirt3 and MnSOD, as well as MnSOD activity in airway epithelial cells in concentration and time-dependent manner, suggesting that the Sirt3-MnSOD signaling axis is involved in cigarette smoke-induced airway epithelial cell injury. Sirt3 silencing intensified the decline of MnSOD expression and activity and aggravated mitochondrial oxidative stress and cell injury induced by CSE. In contrast, Sirt3 overexpression protected cells from cigarette smoke-induced damage. Thus, our results are consistent with another study, which showed that Sirt3 is protected against urban particulate matter-induced autophagy and oxidative stress in human bronchial epithelial cells [38]. Our findings improve the understanding of the role of Sirt3 in the pathogenesis of COPD and implicate the contribution of MnSOD as a mediator of tissue protection. However, one limitation of this study is that the levels of MnSOD acetylation was not measured in COPD airway epithelium and in CSE-treated airway epithelial cells. The role of MnSOD acetylation in the mitochondrial damage of the airway epithelium and its contribution to the progression of COPD should be further explored in future studies.

**Figure 7:**
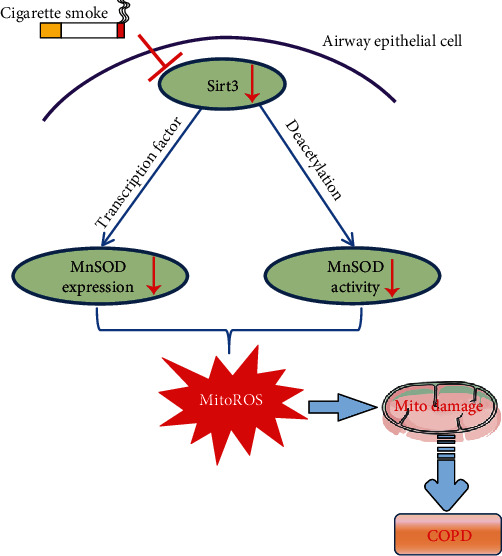
Graphic summary of the protective role of Sirt3 on airway epithelial mitochondrial oxidative injury in COPD. Cigarette smoke exposure decreases Sirt3 expression in airway epithelial cells, thereby contributing to the mitochondrial damage and COPD pathogenesis through the regulation of MnSOD expression and activity. ⊥: inhibition, ↓: decline.

In conclusion, we have demonstrated that Sirt3 inhibited mitochondrial oxidative stress in airway epithelial cells possibly by the upregulation of MnSOD. After the cigarette smoke exposure, Sirt3 expression in airway epithelial cells is significantly decreased and reduces both MnSOD expression and activity. This process contributes to mitochondrial damage and the pathogenesis of COPD as summarized in Figure 7. Therefore, activating the Sirt3-MnSOD signaling pathway might present a novel therapeutic target to slow or prevent the pathogenesis of COPD.

## Data Availability

The data used to support the findings of this study are included within the article.
